# Monitoring and driving factors analysis of vegetation in Henan Province from 2000 to 2023

**DOI:** 10.1371/journal.pone.0357044

**Published:** 2026-08-27

**Authors:** Junqiang Xu, Wendi Luo, Chao Ren, Lei Wang, Yafen Yu, Qian Yang

**Affiliations:** 1 College of Geo-Exploration Science and Technology, Jilin University, Changchun, China; 2 Henan First Geology and Mineral Survey Institute Co., Ltd., Zhengzhou, China; 3 School of Geomatics and Prospecting Engineering, Jilin Jianzhu University, Changchun, China; 4 Jilin Institute of GF Remote Sensing Application, Changchun, China; 5 Northeast Institute of Geography and Agroecology, Chinese Academy of Sciences, Changchun, China; Zhejiang Agriculture and Forestry University: Zhejiang A and F University, CHINA

## Abstract

Vegetation dynamics reflect coupled climate and land-use change, yet their drivers often vary across physiographic and basin contexts, complicating regional attribution. This study assesses spatiotemporal NDVI changes in Henan Province, China, from 2000 to 2023 and diagnoses the relative roles of climate and human activities across the Yellow, Huai, Hai, and Yangtze river basins. Annual mean growing-season NDVI time series were analyzed using Theil–Sen slope estimation and significance testing to map trend magnitude and direction. Basin-scale climate–vegetation relationships were examined with partial correlation against temperature and precipitation, while GeoDetector was used to quantify dominant controls and interaction effects among topographic and edaphic factors. A residual trend approach was further applied to separate the net anthropogenic signal after accounting for climatic influences, and pixels with statistical significance (p < 0.05) were mapped. Results indicate an overall greening tendency with marked spatial heterogeneity, including localized degradation hotspots. Topography and soil-related factors and their interactions explain substantial spatial stratified heterogeneity, whereas NDVI responses to temperature and precipitation differ among basins. Residual attribution suggests that human activities predominantly promote vegetation improvement, while inhibiting effects are more localized and fragmented. These findings provide basin-explicit evidence for targeted ecosystem management and climate-adaptive land-use planning in Henan.

## 1. Introduction

Vegetation dynamics serve as sensitive indicators of global environmental change, with their variations directly influencing carbon cycles, biodiversity, and regional sustainable development. This has become a central topic in ecological security and climate change research [1,2]. The state of vegetation growth in areas characterized by intensive human activities is crucial not only for the stability and service functions of local ecosystems but also holds significant scientific value for understanding ecosystem response mechanisms under the interactions between high-intensity human activities and natural processes [3]. However, existing research has predominantly focused on macro scales or key ecological regions. There remains a relative lack of long-term and systematic monitoring of vegetation change and multi-factor driver analysis for areas with pronounced human-land interactions, particularly studies that reveal the relative contributions of natural and anthropogenic factors from the perspective of differences in physiographic units [4,5]. Therefore, systematically elucidating the spatiotemporal characteristics of vegetation change over long time series and its dominant driving factors is of great theoretical and practical significance for deepening the understanding of ecosystem evolution patterns within the Anthropocene context and supporting regional ecological protection, restoration, and adaptive management.

Satellite-derived vegetation indices, particularly the Normalized Difference Vegetation Index (NDVI), have been extensively employed to monitor vegetation activity across spatial and temporal scales due to their strong correlation with photosynthetic capacity and biomass [6]. Long-term NDVI trend analysis, supported by non-parametric statistical methods such as the Theil-Sen estimator and Mann-Kendall test, has become a standard approach for detecting greening or browning trends and assessing their significance [7,8]. Most existing studies in the region have focused on either short-term trends or specific ecological processes (e.g., carbon storage, water consumption) [9,10]. Few have provided a continuous, long-term (>20-year) analysis of vegetation greenness that captures the full trajectory of change since the early 21st century. This limits the understanding of decadal trends and the detection of potential turning points in vegetation activity.

While climate and human activities are recognized as dominant drivers, their relative contributions and interactions at a finer spatial scale—particularly across distinct river basins with heterogeneous environmental and socio-economic settings—remain poorly quantified. Prior basin-level studies in Henan have primarily addressed ecosystem services or land use [11], but have not systematically compared the differential controls of natural factors (topography, soil) and anthropogenic forcing on vegetation trends among the four major river basins (Yellow, Huai, Yangtze, and Hai). This gap hinders the development of basin-specific ecological management strategies.

To attribute vegetation changes to specific drivers, researchers increasingly employ multivariate statistical frameworks and spatial analysis tools. Partial correlation analysis helps isolate the independent association between vegetation and climatic variables by controlling for multicollinearity [12]. Furthermore, the residual trend method, which removes the climatic signal from observed NDVI series, is widely used to quantify the net effect of human activities [13]. More recently, the Geodetector model has gained prominence for its ability to quantify the explanatory power of individual factors and their interactions on spatial heterogeneity, offering a robust tool for spatial stratified heterogeneity analysis [14, 15]. In China, numerous studies have applied these methodologies to assess vegetation dynamics in ecologically sensitive regions such as the Loess Plateau and Tibetan Plateau [16, 17]. However, within the complex transitional landscape of Henan—characterized by intersecting climate zones, diverse topography, and intense human pressure—a comprehensive, long-term, and spatially explicit analysis of vegetation trends and their multifactorial drivers is still lacking.

As a pivotal agricultural and populous province in central China, Henan Province exemplifies a region where rapid socio-economic development intensively interacts with a vulnerable ecological base, making it a critical hotspot for understanding the complex drivers of vegetation change under coupled natural-human systems. To address these gaps, this study aims to conduct a comprehensive monitoring and driving forces analysis of vegetation changes in Henan Province from 2000 to 2023. Specifically, our objectives include: (1) To quantify the spatiotemporal trends of mean growing-season NDVI using the Thei-Sen slope estimator and Mann Kendall test, and to identify regions of significant greening or degradation; (2) to analyze the influence factors on heterogeneity of NDVI trends across the four major river basins; (3) to assess the relative contributions of human activities to vegetation changes.

## 2. Materials and methods

### 2.1. Study area

**Fig 1:**
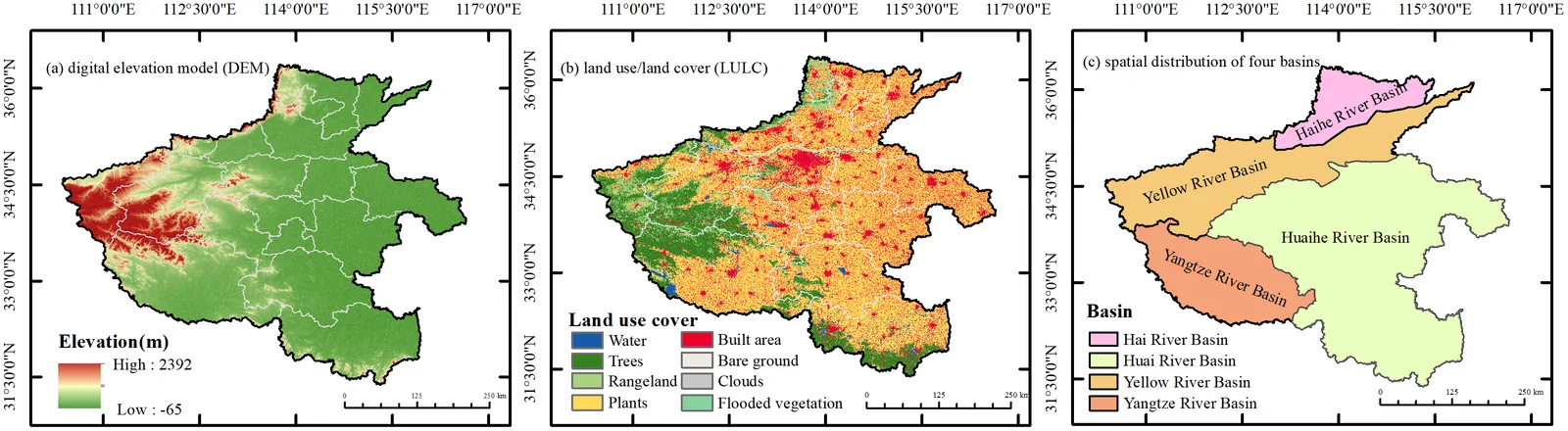
The geographic location of study area. Panel (a) shows the digital elevation model (DEM) illustrating the topographic variation across the region. Panel (b) presents the land use/land cover (LULC) classification, highlighting the distribution of vegetation, water bodies, and urban areas. Panel (c) depicts the spatial distribution of the four major river basins, delineating the Yellow River, Huaihe River, Yangtze River, and Haihe River basins. Base map: Base map layers and administrative boundaries derived from the National Geomatics Service Platform (Tianditu), with official surveying and mapping approval number GS(2024)0650.

Henan Province is located in central–eastern China, along the southern margin of the North China Plain, in the middle–lower reaches of the Yellow River. It lies between 31°23′–36°22′ N and 110°21′–116°39′ E [18]. The province borders Shandong and Anhui to the east, Shaanxi to the west, Hebei and Shanxi to the north, and Hubei to the south. The location and administrative division of Henan Province are shown in Fig 1. Henan spans approximately 530 km from north to south and 580 km from east to west, with a total area of about 167,000 km^2^. The climate is predominantly warm temperate, with the southern part extending into the subtropics, representing a transitional continental monsoon climate from the northern subtropics to the warm temperate zone. Influenced by the east–west topographic gradient, the climate also exhibits a transition from plains to hills and mountains, characterized by distinct seasons, synchronous rainfall and heat, diverse climatic conditions, and relatively frequent meteorological hazards. The long-term mean annual temperature ranges from 15.1 to 15.9 °C, mean annual precipitation from 512.6 to 1129.1 mm, and mean annual sunshine duration from 1774.5 to 2024.1 h [7]. Hydrologically, Henan belongs to four major river including the Yellow, Huai, Yangtze, and Hai river basins, and the study area was therefore divided into four basins for zonal analyses [9,19].

### 2.2. Remote sensing data

This study used the MODIS NDVI product (MOD13A3, monthly composite, 1-km resolution). Each pixel in the MOD13A3 product is accompanied by a Quality Control (QC) flag that indicates pixel reliability. To ensure data quality, we retained only pixels flagged as high quality and acceptable quality, while discarding pixels marked as cloud, snow/ice, or data missing. In addition, a water mask was applied to exclude water bodies (e.g., rivers, lakes, reservoirs) from the analysis, preventing aquatic pixels from confounding the vegetation signal. The raw NDVI values, stored as scaled integers, were converted to physical values by multiplying by 0.0001 according to the MODIS product specification. After quality filtering and spatial preprocessing (reprojection and resampling to 1-km resolution), we calculated the mean growing-season NDVI (NDVI_GS_) for each year by averaging monthly NDVI values from June to September. Pixels with missing or invalid values in any given month were excluded from the seasonal averaging, ensuring that the resulting annual NDVI_GS_ time series (2000–2023) contained only valid observations for trend analysis and subsequent attribution. NDVI_GS_ represents vegetation greenness during the main growing season and is less sensitive to occasional outliers than annual maximum NDVI. This approach effectively mitigates the impacts of disturbances such as clouds and aerosols, ensuring the scientific validity and reliability of the vegetation growth assessment.

### 2.3. Influence factors

Based on the GeoDetector model, two meteorological factors, two soil factors, and three topographic factors were used to analyze their influence on the spatiotemporal distribution characteristics of vegetation (Table 1). To ensure spatial scale consistency, all auxiliary data were resampled to 1-km resolution in either ArcGIS or the GEE platform. For categorical data (e.g., soil type), the nearest neighbor method was applied; while for continuous data (e.g., elevation, slope, aspect), the bilinear interpolation method was used.

**Table 1:** The data source used in this study.

Type	Data name	Temporal resolution	Spatial resolution	Data source
Vegetation index	MODIS NDVI	Monthly	1 km	https://code.earthengine.google.com/
Meteorological factors	Temperature	Monthly	1 km	https://www.geodata.cn/main/
	Precipitation	Monthly	1 km	https://www.geodata.cn/main/
Soil factors	Soil type		30 m	https://www.resdc.cn
	Soil moisture	Daily	1 km	https://data.tpdc.ac.cn/home
Topographical factors	Elevation	/	30 m	https://www.nasa.gov/
	Slope		30 m	https://www.nasa.gov/
	Aspect		30 m	https://www.nasa.gov/

#### 2.3.1. Meteorological data.

The meteorological data were obtained from the National Earth System Science Data Center [20]. This dataset includes monthly precipitation and temperature data at a 1-km spatial resolution. It was generated by downscaling the global 0.5° climate data from the Climatic Research Unit (CRU) and the high-resolution climate data from WorldClim using the Delta spatial downscaling scheme. The measurement accuracy for precipitation and temperature is 0.1 mm and 0.1 °C, respectively.

#### 2.3.2. Soil data.

The soil type data were obtained from the Harmonized World Soil Database (HWSD) version 1.1, released in 2009 by organizations including the Food and Agriculture Organization of the United Nations, the International Institute for Applied Systems Analysis, ISRIC – World Soil Information, and the Chinese Academy of Sciences. This database integrates regional and national soil information from around the world, with a spatial resolution of 1-km. The soil classification follows the FAO-90 system, and common soil types in the study area include Fluvisols, Regosols, and Anthrosols, among others. The soil moisture data were obtained from the National Tibetan Plateau/Third Pole Environment Data Center [21]. This dataset was generated using a machine learning approach, with 10-layer soil moisture measurements from 1,648 stations operated by the China Meteorological Administration serving as the benchmark. The covariates for model training included ERA5_Land meteorological forcing data, leaf area index (LAI), land cover type, digital elevation model (DEM), and soil properties. The data unit is 0.001 m^3^/m^3^.

#### 2.3.3. Topographic data.

The topographic data were derived from the SRTM 30 m dataset, jointly released by agencies including the National Aeronautics and Space Administration (NASA) and the National Imagery and Mapping Agency (NIMA). Elevation, slope, and aspect data for the study area were extracted from this dataset for further analysis.

### 2.4. Methods

#### 2.4.1. Trend analysis.

This study comprehensively applied the Theil-Sen median slope estimator and the Mann-Kendall non-parametric test to analyze vegetation change trends and examine their significance, thereby quantifying the spatiotemporal dynamics of vegetation cover in the study area from 2000 to 2023. The Theil-Sen slope estimator, proposed by Henri et al., is effective in handling outliers and missing values [22,23]. In this study, the Theil-Sen median slope was employed to calculate the NDVI_GS_ time-series trend. This method calculates the median of slopes from all possible point pairs in the time series, establishing a statistically robust trend characterization framework. A slope greater than 0 indicates an increasing trend in vegetation coverage, while a slope less than 0 signifies a decreasing trend. This was combined with the non-parametric Mann Kendall (MK) test [24,25] to assess trend significance. This test does not assume a specific data distribution and detects monotonic trends by constructing a rank sequence. Significance was determined at the α = 0.05 level.

#### 2.4.2. Analysis of spatial driving forces.

Partial correlation analysis at the pixel scale was employed to eliminate interference from multicollinearity among variables and to quantitatively assess the independent association between NDVI_GS_ and climatic factors. The monotonic relationship was measured using Spearman’s rank correlation coefficient [26–29], and the statistical reliability of the correlation was determined through significance testing (p < 0.05).

This study quantified the components of vegetation dynamics not explained by climatic factors by constructing a trend analysis framework based on residual sequences. First, the linear influences of temperature, precipitation, and soil moisture were removed from the observed NDVI_GS_ values to generate a standardized residual series. Subsequently, the long-term trend of residual changes was estimated based on the median slope, and the statistical significance of the trend was verified using the Mann-Kendall test.

The Geodetector [30–32] was employed to quantify the explanatory power of the factors, which provides a quantitative assessment of a factor’s explanatory power over the target variable. The calculation is listed as below:


$$\begin{array}{c} 𝐪=1-\frac{\sum_{h=1}^{L}N_{h}{\sigma_{h}}^{2}}{N\sigma^{2}} \end{array}$$ (1)

Where L is the number of regional classifications; Nh represent the sample size in the h-th stratum; ${\sigma_{\text{h}}}^{\text{2}}$ denotes the variance of the target variable in the h-th stratum; N is the total sample size in the study area; $\sigma^{\text{2}}$ is the overall variance of the target variable across the study area. GeoDetector requires explanatory variables to be formatted as categorical strata. For the continuous variables elevation and slope, we discretized them into 6 classes using the Jenks Natural Breaks method, which minimizes within-class variance while maximizing between-class variance, thereby identifying statistically meaningful thresholds in spatially continuous topographic data. The number of classes was set to k = 6 after testing alternative class numbers (k = 4, 5, 7, 8); the dominant factors and their interaction patterns remained stable at this level, indicating that k = 6 captures the essential spatial stratification without introducing excessive fragmentation. For aspect, we categorized it into 8 directional classes (N, NE, E, SE, S, SW, W, NW) following standard cartographic convention. Soil type was classified into 6 categories based on the FAO-90 classification system adopted in the HWSD database.

This study quantified the components of vegetation dynamics that remained unexplained by climatic factors by performing trend analysis on the residual series. First, linear components influenced by temperature, precipitation, and soil moisture are removed from the observed NDVI_GS_ values to generate standardized residual series. Subsequently, the long-term trend of residual changes is estimated based on the median-based slope, and the statistical significance of the trend is assessed using the Mann Kendall test. This dual robust statistical strategy ensures both the reliability of trend estimation and provides a quantitative basis for identifying non-climatic drivers, such as human activities [33–35].


$$\begin{array}{c} {NDVI}_{GS}=\beta_{0}+\beta_{1}T_{t}+\beta_{2}P_{t}+\beta_{3}S_{t}+\epsilon_{t} \end{array}$$ (2)

where, Tt, Pt, and St represent observed temperature, precipitation, and soil moisture, respectively; β0 is the intercept term; β1, β2, and β3 are regression coefficients; εt is the residual term, representing NDVI_GS_ changes unexplained by climatic factors; and t is the time series index, denoting a specific year.

## 3. Results

### 3.1. Spatial changes of NDVI_GS_

**Fig 2:**
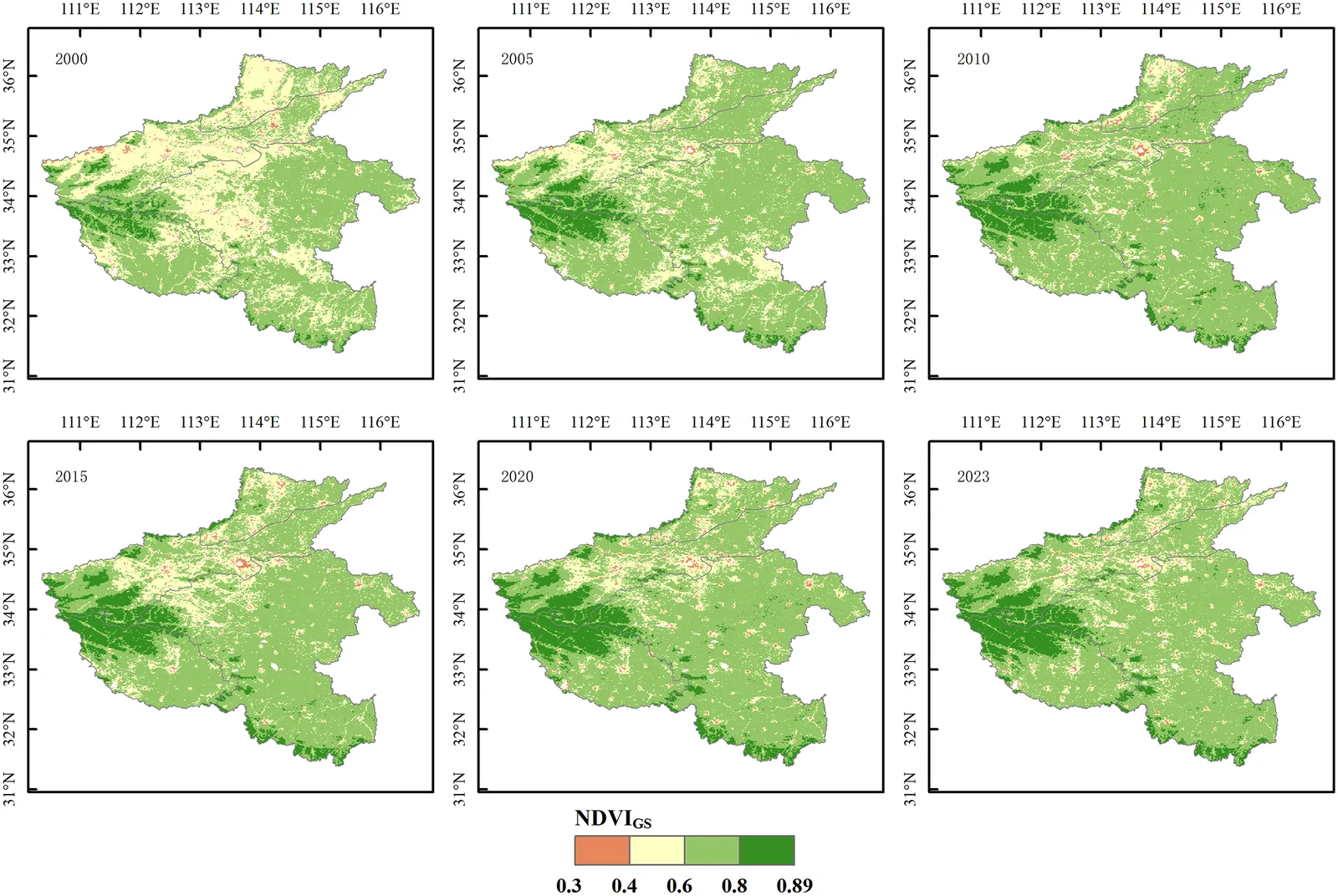
The spatial distribution of NDVI_GS_ across Henan Province from 2000 to 2023. Sequential maps illustrate the spatial distribution and interannual variations of NDVI_GS_ during the study period. The color gradient represents NDVI values ranging from 0.3 to 0.89, highlighting the seasonal phenology and overall vegetation trends in the region. Base map: Base map layers and administrative boundaries derived from the National Geomatics Service Platform (Tianditu), with official surveying and mapping approval number GS(2024)0650.

Fig 2 displays the spatial distribution of the mean growing season Normalized Difference Vegetation Index (NDVI_GS_) from 2000 to 2023, with the results presented at five-year intervals. According to the analysis of multi-year spatial distribution of NDVI_GS_, its high-value and low-value areas exhibit stable and recurring spatial patterns. The high-value NDVI_GS_ areas consistently concentrate in two regions with superior natural ecological conditions: first, the junction zone of Luoyang, Sanmenxia, and Nanyang in the west, which is dominated by mountainous and hilly terrain such as the Funiu Mountains, featuring lush natural vegetation and minimal human disturbance; second, the southern region of Xinyang, benefiting from its subtropical climate south of the Huai River and mountainous environment, where vegetation coverage remains consistently high. In stark contrast, the low-value NDVI_GS_ areas persistently recur in the central region centered on Zhengzhou, primarily due to rapid urbanization and dense expansion of construction land, which significantly compress natural vegetation space and result in continuously low vegetation indices in this area. Overall, this spatial distribution pattern profoundly reflects the supporting role of natural geographical foundations on vegetation coverage and the persistent impact of intense human activities on surface vegetation.

**Fig 3:**
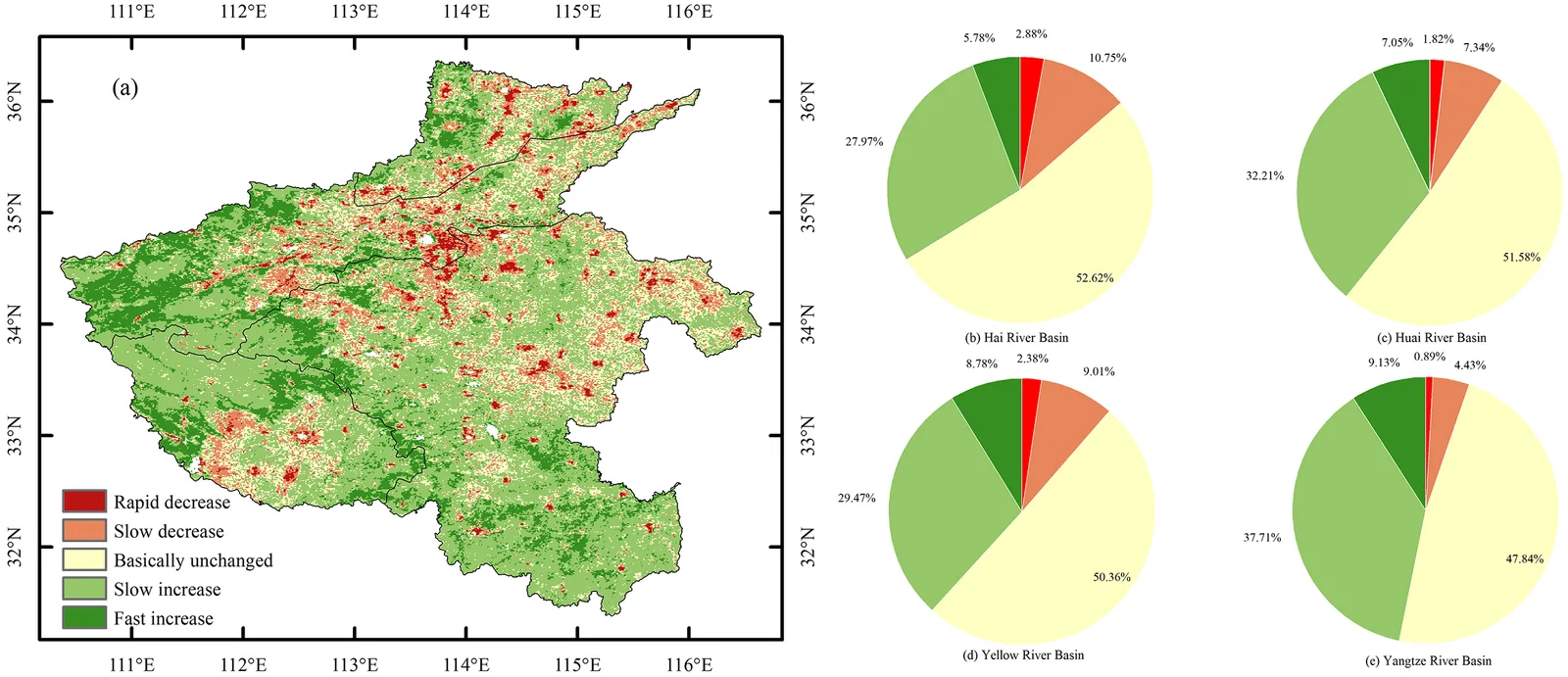
Changing trend of the NDVI_GS_ across Henan Province from 2000 to 2023 based on Theil-Sen estimator. Panel (a) presents the spatial distribution of NDVI_GS_ trends, classified into five categories: rapid decrease (red), slow decrease, basically unchanged, slow increase, and fast increase. Panels (b – e) illustrate the proportional composition of these change categories within the Haihe, Huaihe, Yellow, and Yangtze River basins, respectively. Base map: Base map layers and administrative boundaries derived from the National Geomatics Service Platform (Tianditu), with official surveying and mapping approval number GS(2024)0650.

Fig 3 illustrates the spatial distribution of trends in NDVI_GS_ across Henan Province from 2000 to 2023. Based on the significance test (p-value), vegetation change was classified into five categories: rapid decrease, slow decrease, basically unchanged, slow increase, and rapid increase. According to the Theil–Sen slope estimates, vegetation dynamics in the study area were dominated by greening. Areas with increasing NDVI_GS_ accounted for 66.50% of the province, including 52.28% with a slow increase and 14.22% with a rapid increase, corresponding to a total greening area of approximately 11.106 × 104 km^2^. In addition, 19.50% of the area remained basically unchanged. In contrast, areas with decreasing NDVI_GS_ represented only 14.00% of the province, comprising 11.22% with a slow decrease and 2.78% with a rapid decrease. Spatially, pixels exhibiting increasing NDVI_GS_ were widespread and formed a relatively continuous improvement belt in western–southern Henan. By contrast, pixels with decreasing NDVI_GS_ were more fragmented and patchier, concentrating mainly in the central–eastern plains and around urban areas, which is consistent with the effects of built-up land expansion and intensified land use. Overall, vegetation cover in Henan Province has improved markedly over the past two decades; however, localized degradation hotspots warrant further investigation of underlying drivers and targeted management to mitigate potential ecological risks.

When disaggregated by major river basins, all subregions still exhibited an overall greening tendency, but the magnitude differed. The Yangtze River Basin showed the strongest greening of with 46.84% pixels increasing and the lowest degradation with 5.32% pixels decreasing, whereas the Hai River Basin had the highest proportion of declining pixels (13.63% decrease) and the largest share of basically unchanged areas (52.62%). The Huai and Yellow River basins were intermediate, with 39.26% and 38.25% of pixels increasing, respectively, and relatively limited decreases (9.16% and 11.39%). Overall, vegetation cover in Henan Province has improved markedly over the past two decades; however, localized degradation hotspots warrant further investigation of underlying drivers and targeted management to mitigate potential ecological risks.

### 3.2. The influence of soil and topography on vegetation

**Fig 4:**
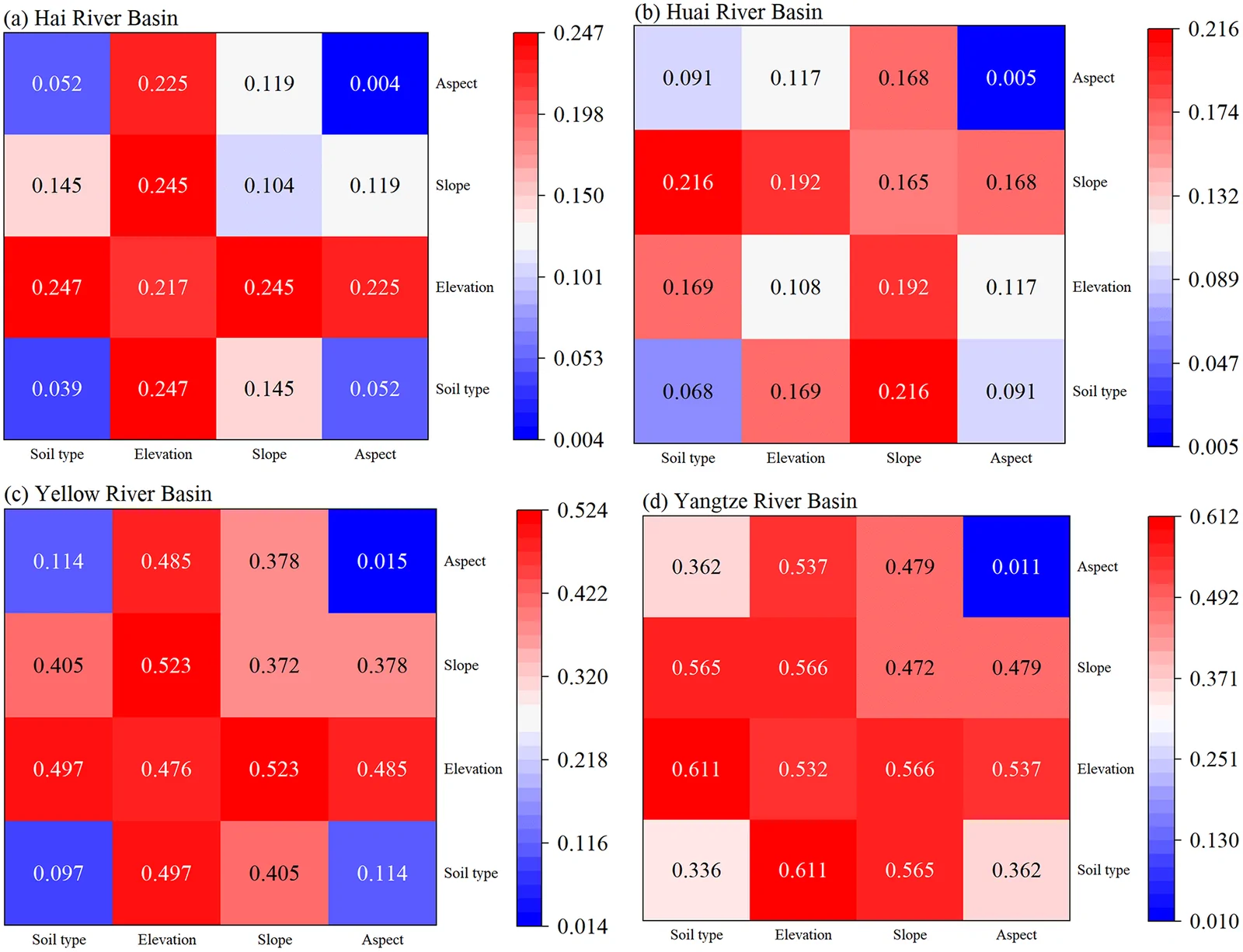
The GeoDetector-based factor and interaction effects on NDVI_GS_ trend variability across four major river basins in Henan Province.

Fig 4 reveals the dominant factors driving the spatial heterogeneity of NDVI_GS_ trends based on q-values of GeoDetector, across the four major river basins and their interaction effects. Overall, topographic factors (elevation and slope) generally exhibit stronger explanatory power than aspect, and most two-factor interactions show enhancement effects. Specifically, in the Hai River Basin, elevation is the strongest single factor with a q-values of 0.217, and the strongest interaction occurs between elevation and soil type, with a q-values of 0.247. In the Huai River Basin, slope is the strongest single factor with a q-values of 0.192, and the strongest interaction is between slope and soil type, with a q-values of 0.216. In the Yellow River Basin, the explanatory power of single factors increases markedly; elevation contributes the most with a q-values of 0.476, and the strongest interaction is the elevation–slope interaction, reaching a q-values of 0.523. The Yangtze River Basin shows the highest explanatory power overall; elevation remains the strongest single factor (q = 0.532), while the strongest interaction is between soil type and elevation, with a q-values of 0.611. In general, q-values in the Yellow and Yangtze river basins are clearly higher than those in the Hai and Huai river basins, indicating that the control of natural topography–soil gradients on the spatial pattern of NDVI_GS_ trends is more pronounced in areas with greater topographic relief.

**Fig 5:**
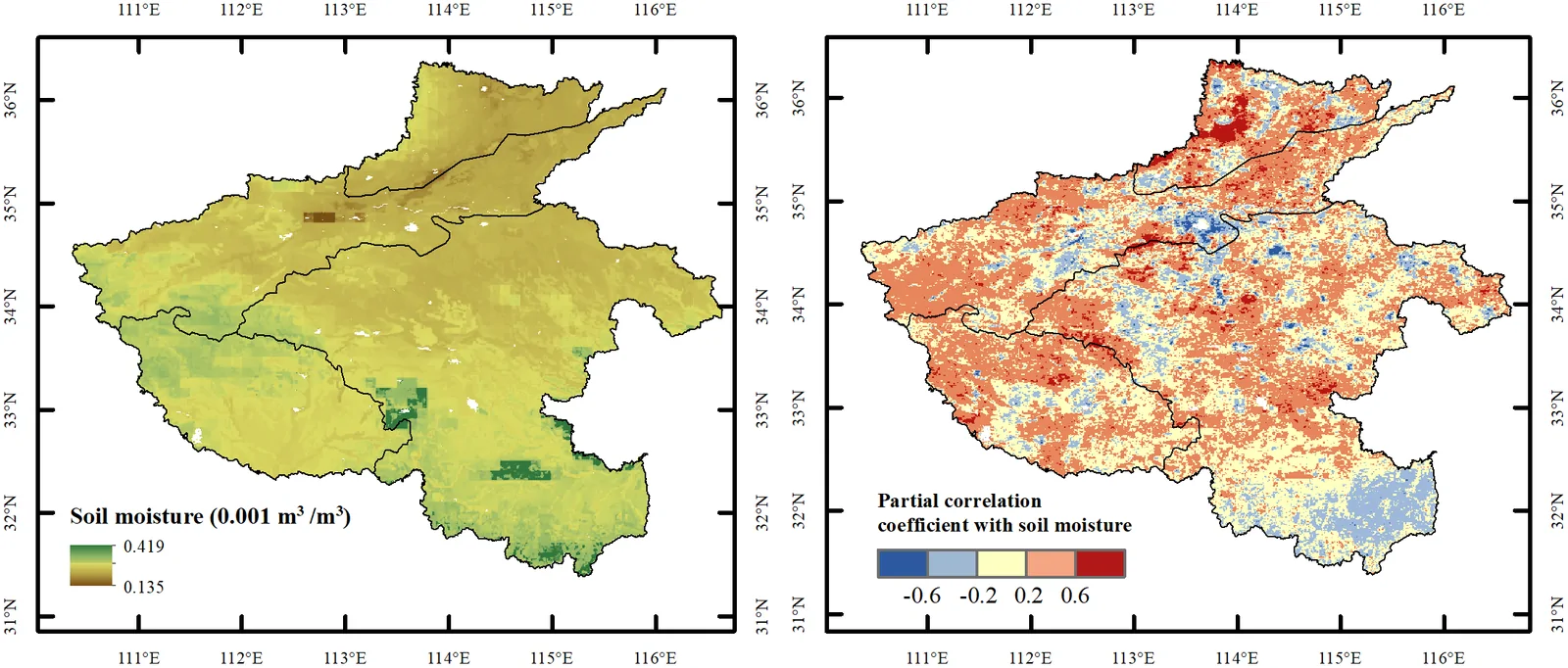
The relationship between soil moisture and NDVI_GS_. Panel (a) illustrates the spatial pattern of surface soil moisture, with the color gradient indicating volumetric water content ranging from 0.135 to 0.419  × 10^-3^ m^3^/m^3^. Panel (b) presents the partial correlation coefficient map between soil moisture and NDVI_GS_, where red tones signify positive correlations and blue tones indicate negative correlations. Base map: Base map layers and administrative boundaries derived from the National Geomatics Service Platform (Tianditu), with official surveying and mapping approval number GS(2024)0650.

In Henan Province, the spatial distribution of soil moisture exhibits a distinct southeast-to-northwest decreasing gradient (Fig 5a). The lower Huai River basin and adjacent areas near the Yangtze River region in the southeast benefit from relatively abundant precipitation and dense river networks, resulting in generally higher soil moisture levels. In contrast, the Yellow River basin and Hai River basin in the northwest are influenced by a semi-arid climate, leading to significantly lower soil moisture. Partial correlation analysis indicates (Fig 5b) that soil moisture is significantly positively correlated with NDVI_GS_ across most of the study area, confirming that water availability is a major environmental factor influencing vegetation growth in this region. Areas with high partial correlation coefficients are mainly concentrated in parts of the Huang-Huai-Hai Plain and the middle Yangtze River region. These regions exhibit soil moisture conditions near ecological thresholds, with frequent seasonal drought events, making vegetation growth highly sensitive to changes in soil moisture and thus representing typical ecological transition zones. Low partial correlation coefficients are primarily observed in two types of areas: the perennially high-moisture regions in the southeast, where water is no longer a limiting factor for vegetation growth, and the arid northwestern regions, where extreme water scarcity coupled with other environmental stressors (such as high evaporation) weakens the statistical relationship between soil moisture and vegetation growth. Furthermore, in topographically complex mountainous areas such as the Funiu Mountains, the partial correlation coefficients display pronounced patchy spatial heterogeneity, reflecting the regulatory effects of local topography, soil properties, and vegetation types on the moisture‑vegetation relationship and highlighting the complexity of small-scale eco-hydrological processes in mountainous regions.

### 3.3. The influence of climate change on vegetation

**Fig 6:**
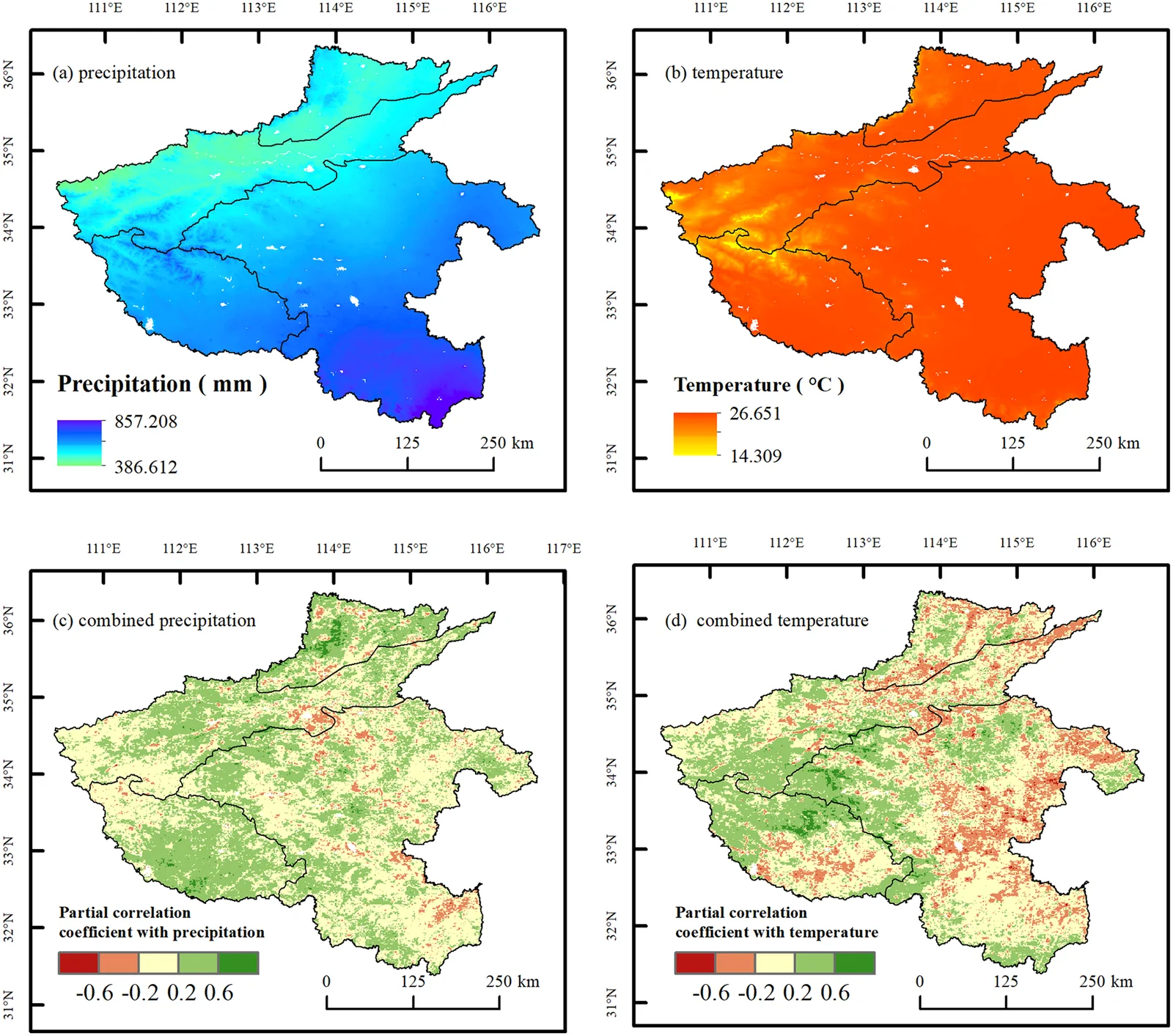
The partial correlation between climate and NDVI_GS_. Panel (a) illustrates the spatial distribution of annual precipitation, with values ranging from 386.612 mm to 857.208 mm. Panel (b) presents the spatial pattern of mean annual air temperature, ranging from 14.309°C to 26.651°C. Panel (c) and Panel (d) depict the partial correlation coefficients between precipitation and NDVI_GS_, and temperature and NDVI_GS_, respectively. Base map: Base map layers and administrative boundaries derived from the National Geomatics Service Platform (Tianditu), with official surveying and mapping approval number GS(2024)0650.

Fig 6 displays the spatial patterns of precipitation and temperature across Henan Province and the partial-correlation responses of NDVI_GS_ to these two climatic factors, highlighting clear basin-level differences. Precipitation generally exhibits a gradient from lower values in the north–northwest to higher values in the south–southeast, with a range of 386.612-857.208 mm; at the basin scale, the Hai River Basin in the north and the Yellow River Basin in the northwest are characterized by relatively low-precipitation conditions, whereas the Huai River Basin in the central–eastern region and the Yangtze River Basin in the south receive comparatively higher precipitation. Temperature shows a strong topographic control, ranging from 14.31 to 26.65 °C, with lower temperatures in the western mountainous areas (Taihang–Funiu Mountains) and higher temperatures over the plains; correspondingly, the mountainous headwaters of the Yellow and Yangtze river basins tend to form low-temperature belts, while the plains of the Hai–Huai river basins are generally warmer.

The partial correlation between NDVI_GS_ and precipitation in Fig 6(c) is dominated by weak to moderate positive relationships, but notable basin contrasts are evident: positive-correlation patches are more common in the relatively water-limited Hai and Yellow river basins, indicating stronger water constraints and greater NDVI_GS_ sensitivity to increased precipitation, whereas weak or negative correlations are more concentrated in the Huai River Basin plains where precipitation is more abundant and agricultural and engineering regulation is stronger, suggesting that the dominant role of precipitation may be partly weakened by irrigation, multiple-cropping systems, drainage conditions, and land-use change.

The partial correlation between NDVI_GS_ and temperature in Fig 6(d) shows an even clearer basin contrast: negative correlations are more widespread across the Hai and Huai plains, implying that warming may suppress vegetation growth by enhancing evapotranspiration and heat stress and thereby aggravating water deficits; in contrast, positive correlations are more prevalent in the mountainous/hilly areas of the Yellow and Yangtze river basins, indicating that under relatively cool conditions, warming is more likely to promote vegetation growth by extending the growing season and increasing photosynthetic potential. Overall, these patterns suggest that NDVI_GS_ responses to climate are jointly regulated by basin-scale hydrothermal backgrounds and topographic conditions.

### 3.4. The human activities on vegetation

**Fig 7:**
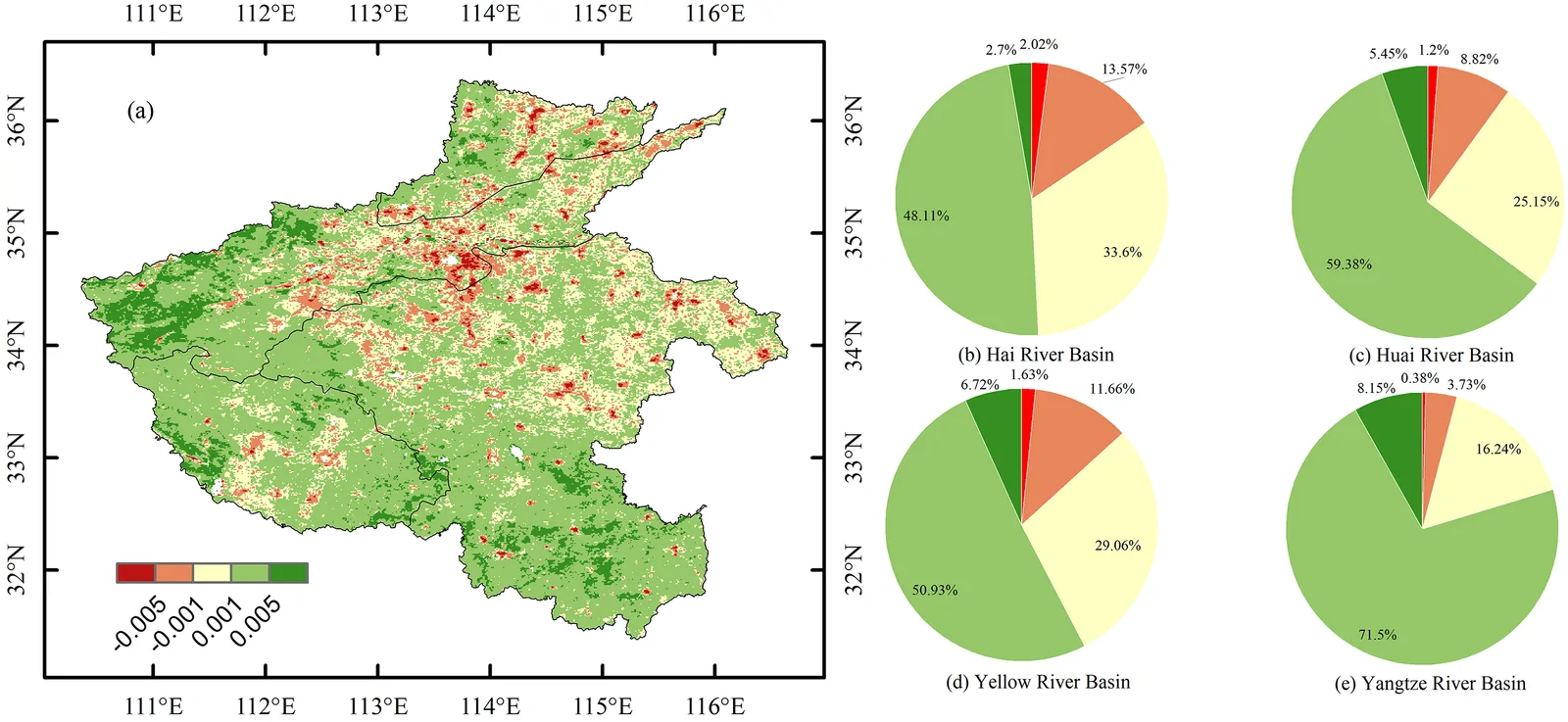
The effects of human activities on NDVI_GS_ changes in Henan Province using a residual trend approach. Panel (a) illustrates the spatial distribution of the linear regression coefficients of NDVI_GS_ against time. Positive values indicate regions of vegetation greening, while negative values denote browning trends. Panels (b–e) present the proportional composition of these temporal trends within the Haihe, Huaihe, Yellow, and Yangtze River basins, respectively, highlighting the dominant role of human activities in driving regional vegetation changes. Base map: Base map layers and administrative boundaries derived from the National Geomatics Service Platform (Tianditu), with official surveying and mapping approval number GS(2024)0650.

Fig 7 quantifies the independent effects of human activities on NDVI_GS_ changes in Henan Province using a residual trend approach after removing the influences of climate and other natural factors. The residual slopes for 2000–2023 are classified into five levels based on the following thresholds: rapid decrease for slopes <−0.005, slow decrease for −0.005 to −0.001, basically stable for −0.001 to 0.001, slow increase for 0.001 to 0.005, and rapid increase for slopes > 0.005. At the provincial scale, areas with positive effects (i.e., positive residual trends, including slow increase and rapid increase) dominate, showing a wider and more spatially continuous distribution, whereas areas with negative effects (i.e., negative residual trends, including slow decrease and rapid decrease) are more patchy and fragmented, indicating that the promoting effects of human activities are more prevalent overall, although localized inhibition or degradation hotspots still exist.

Basin-level comparisons further reveal clear heterogeneity and a distinct ranking of human-activity effects. The Yangtze River Basin exhibits the strongest positive effects, with 71.50% under slow increase and 8.15% under rapid increase (79.65% in total), and it also has the lowest weakened-effect area at 4.26%. The Huai River Basin shows 64.87% positive effects and 10.02% negative effects, while the Yellow River Basin has 57.71% positive effects and 13.29% negative effects. In contrast, the Hai River Basin shows the weakest positive effects at 50.81% but the highest negative effects at 15.59%, and it also has the largest share of basically stable areas at 33.60%. Overall, the net effect of human activities is predominantly promoting, but a pronounced basin-scale gradient is evident (Yangtze > Huai > Yellow > Hai), highlighting strong spatial heterogeneity in the independent influence of human activities on NDVI_GS_.

**Fig 8:**
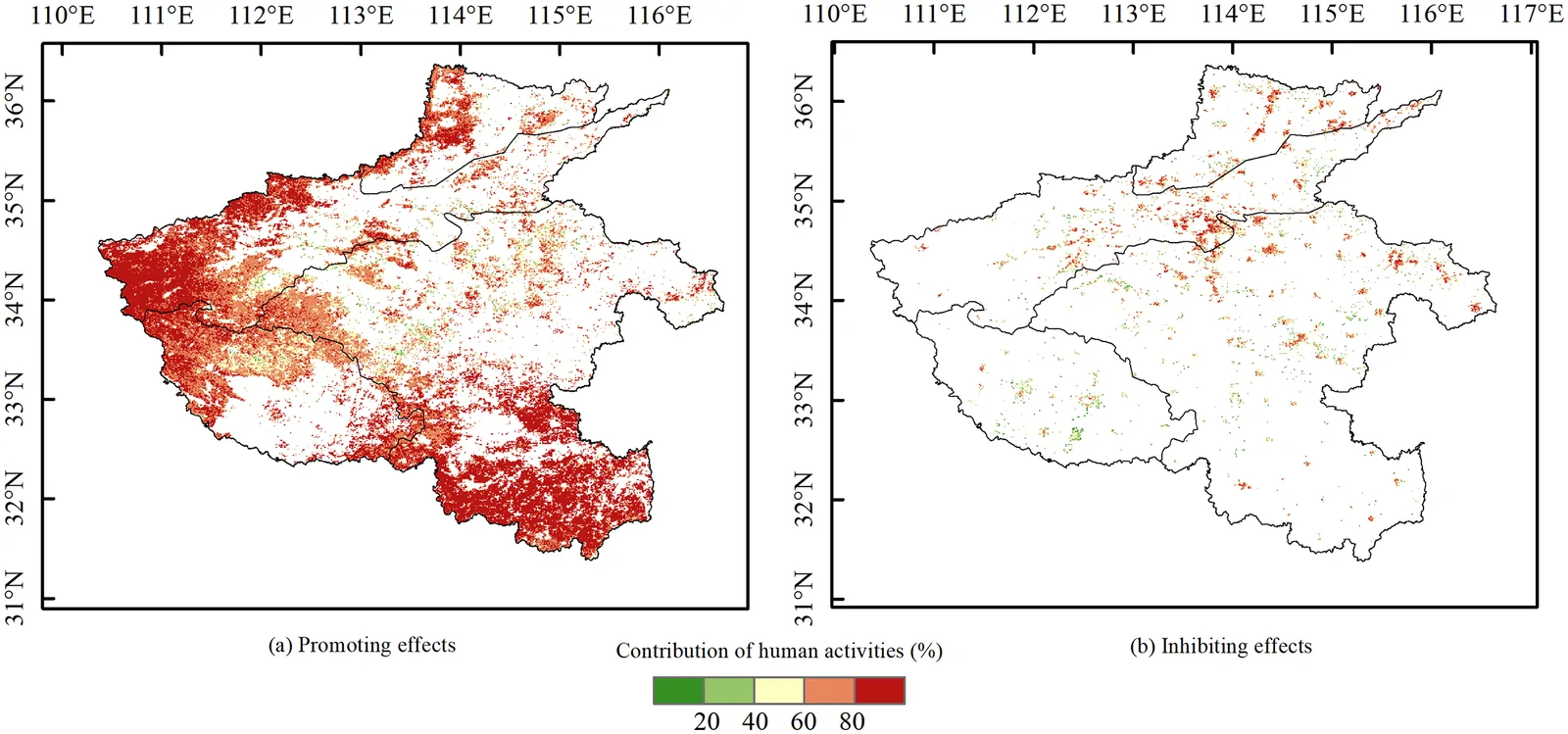
The effects of human activities on NDVI_GS_ changes in Henan Province using a residual trend approach. Panel (a) illustrates the spatial distribution of the promoting effects of human activities, represented as the percentage contribution of human activities to vegetation greening. Panel (b) shows the spatial pattern of the inhibiting effects, indicating the percentage contribution of human activities to vegetation browning. The color gradient indicates the magnitude of the contribution percentage. Base map: Base map layers and administrative boundaries derived from the National Geomatics Service Platform (Tianditu), with official surveying and mapping approval number GS(2024)0650.

A basin-scale comparison indicates pronounced differences in the effects of human activities on NDVI_GS_ among the major basins in Fig 8. The spatial distribution of these net anthropogenic effects is consistent with the spatial pattern of NDVI_GS_ trend changes identified in Section 3.1: the concentrated inhibiting-effect patches closely coincide with the fragmented NDVI-decreasing pixels that were found to be mainly distributed over the central-eastern plains and around urban areas, whereas the more continuous promoting-effect belts align with regions of sustained vegetation improvement in western-southern Henan.

Specifically, the Yangtze River Basin, located in the southern part of the study area and dominated by mountainous and hilly terrain, shows a more continuous distribution of high-contribution areas associated with promoting effects and relatively few inhibition patches, suggesting that human interventions such as ecological restoration and conservation measures play a dominant role in enhancing vegetation in this basin. The Yellow and Huai river basins generally exhibit a pattern of promotion dominance with localized inhibition, and inhibition patches are more concentrated over the plains of the Huai River Basin, implying that negative disturbances linked to intensive land use — such as urban expansion and high-intensity agricultural development — are more likely to emerge in these areas. The Hai River Basin in the north is characterized by comparatively weaker promoting effects and more numerous, highly fragmented inhibition hotspots, indicating that in regions with higher development intensity, NDVI_GS_ is more sensitive to anthropogenic disturbance and inhibition effects are more likely to dominate locally.

Overall, all four basins display strong spatial heterogeneity: promoting effects tend to form relatively continuous high-contribution belts in areas with better ecological backgrounds or where ecological projects are concentrated, whereas inhibiting effects closely coincide with zones of intensive human disturbance and appear as localized, fragmented patches. This spatial correspondence between net anthropogenic effects and observed NDVI_GS_ trends reinforces the interpretation that the detected degradation hotspots are largely driven by localized anthropogenic pressures.

## 4. Discussion

This study provides a comprehensive, 24 years assessment of vegetation dynamics and their multifactorial drivers in Henan Province from 2000 to 2023. Our key findings reveal a province-wide greening trend dominated by significant vegetation improvement, alongside pronounced spatial heterogeneity strongly structured by major river basins. Critically, we demonstrate that the relative importance of natural (topography, soil, climate) and anthropogenic drivers varies substantially across these basins, with topographic control predominating in mountainous southwest (Yangtze Basin) and human activities exhibiting a stronger, yet spatially divergent, net effect across the intensively managed eastern plains. The dominant greening trend in Henan is consistent with the widespread greening of China observed in national and global studies, often attributed to afforestation, agricultural intensification, and climate change [15 36]. However, our basin-zonal analysis advances beyond these broad-scale narratives.

The exceptionally high explanatory power (q-values) of elevation and its interactions with slope and soil type in the Yellow and Yangtze River Basins underscores the fundamental role of physical landscape template in regulating vegetation trends where human pressure is relatively lower [37]. In these mountainous and hilly regions, elevation gradients dictate microclimates (e.g., temperature, moisture) and soil development processes, creating distinct ecological niches [38]. The finding that interaction effects (e.g., elevation×soil type) often surpass individual factor effects aligns with ecological theory that ecosystem properties emerge from complex, non-linear interactions between abiotic factors [15]. This suggests that in topographically complex regions, vegetation resilience or sensitivity to change cannot be predicted by single factors alone, supporting the use of interaction detection tools like Geodetector for nuanced spatial attribution [39].

The contrast in q-values among the four basins further reflects their distinct physiographic settings. In the Yangtze River Basin, the highest q-value for elevation (q = 0.532) and the strongest elevation–soil type interaction (q = 0.611) are consistent with the basin’s mountainous and hilly topography, where steep elevation gradients create strongly differentiated microclimatic and edaphic conditions that shape distinct vegetation niches. In the Yellow River Basin, the dominant elevation–slope interaction (q = 0.523) aligns with the topographically fragmented transitional zone along the Loess Plateau margin, where terrain heterogeneity exerts the most pronounced control on NDVI_GS_ spatial variation. By contrast, the Huai and Hai River basins, which are dominated by flat plains with low topographic relief, exhibit comparatively lower explanatory power from individual topographic factors; in these basins, climate sensitivity (Section 3.3) and anthropogenic impacts (Section 3.4) emerge as more prominent drivers of NDVI_GS_variability. This basin-specific hierarchy of drivers — from topography-dominated in the southwest to climate- and human-dominated in the east — highlights the importance of coupling physiographic context with driver attribution.

While previous regional studies noted the importance of elevation or human activities, few have systematically quantified and compared the shifting hierarchy of drivers across adjacent but environmentally distinct hydrological units. For instance, our finding that topography explains over 50% of the spatial variance in NDVI_GS_ trends in the Yangtze Basin (q = 0.532) contrasts sharply with studies on the North China Plain, where human factors dominate [40]. This underscores that extrapolating driver-response relationships from one physiographic region to another can be misleading.

The contrasting partial correlation patterns between NDVI_GS_ and climatic factors are indicative of divergent hydrothermal constraints. The positive correlation of NDVI_GS_ with precipitation in the drier Hai and Yellow River Basins reflects typical water-limited ecosystems, where vegetation growth is tightly coupled with water availability [12]. Conversely, the prevalence of negative correlations with temperature in the Hai and Huai River plains suggests heat-induced water stress, where increased evapotranspiration may outweigh any potential benefits of a lengthened growing season. The positive temperature- NDVI_GS_ correlation in the cooler western mountains, however, aligns with observations from other high-latitude/elevation ecosystems, where warming alleviates temperature limitations on plant growth [41]. These divergent responses highlight that the sign and strength of climate-vegetation coupling are context-dependent, modulated by baseline climatic conditions and water balance [42].

The residual trend analysis effectively disentangles the net anthropogenic signal, revealing a compelling spatial story: human activities have been a net promoter of greening, but with significant inhibitory hotspots. The strongest promoting effects in the Yangtze Basin likely reflect the cumulative impact of large-scale ecological restoration projects (e.g., Grain-for-Green) and reduced pressure due to rural-urban migration, trends documented in southern China’s mountainous regions [15, 16]. In contrast, the concentration of inhibitory effects in the central-eastern plains, especially within the Hai River Basin, spatially coincides with the core areas of urban agglomeration (e.g., Zhengzhou) and intensive agriculture. This pattern mirrors the “human pressure frontier” where land-use intensification and urban expansion directly displace or degrade natural and semi-natural vegetation [43]. The basin-scale gradient (Yangtze > Huai > Yellow > Hai) in net human effect strength likely reflects a parallel gradient in development intensity and the maturity of ecological compensation policies.

Specifically, the concentrated distribution of inhibiting effects — predominantly over the central-eastern plains and around major urban centers — spatially coincides with the fragmented NDVI-decreasing pixels identified in Section 3.1, jointly delineating the core zones of anthropogenic disturbance driven by built-up land expansion and intensive agricultural land use. Conversely, the relatively continuous promoting-effect belts align with regions of sustained vegetation improvement in western-southern Henan, where ecological restoration interventions (e.g., Grain-for-Green Program, mountain closure for afforestation) have been concentrated since the early 2000s. This spatial alignment within the paper’s own results provides an internally consistent argumentative loop: the residual-based anthropogenic signal is not a generic attribution, but one that can be cross-validated against the independently observed vegetation trend patterns and the known spatial distribution of major human activity types across the province.

Beyond the empirical findings, this study makes three contributions to ecological theory and methodological practice. First, it complements the prevailing “China greening” narrative by demonstrating that the hierarchy of dominant drivers is highly scale- and context-dependent. The basin-resolved analysis reveals a clear spatial gradient — from topography–soil interaction dominance in the mountainous southwest to climate sensitivity and anthropogenic predominance in the intensively managed east — thereby challenging the assumption that single-factor or spatially homogeneous driver explanations can be universally extrapolated across physiographic regions. Second, the finding that interaction effects (e.g., elevation ∩ soil type) consistently surpass individual factor effects in explaining NDVI_GS_ spatial variation provides empirical support for the ecological theory that ecosystem properties emerge from complex, non-linear interactions among abiotic factors, while also offering a methodological exemplar for applying interaction detection tools (e.g., GeoDetector) to spatial attribution problems. Third, the directional reversal of climate–vegetation partial correlations across basins — positive in the cooler, moisture-sufficient western mountains and negative in the warmer, water-limited eastern plains — lends multi-basin comparative evidence to the theoretical proposition that the sign and strength of climate–vegetation coupling are fundamentally modulated by baseline hydroclimatic conditions and water balance. Collectively, these contributions advance the understanding of vegetation dynamics in coupled natural–human systems beyond simple greening–browning dichotomies.

This study has several limitations that point to future research needs. First, the use of 1-km NDVI_GS_ data, while suitable for regional analysis, may miss fine-scale vegetation changes associated with fragmented land uses, particularly in urban–peri-urban interfaces where land-use mosaics change at sub-kilometer scales [44]. This spatial resolution constraint limits our ability to resolve vegetation dynamics at the finest scales, especially in rapidly urbanizing areas where the sharpest vegetation transitions occur. Future work could integrate higher-resolution imagery (e.g., Sentinel-2 or Landsat time series) for hotspot areas to better capture fine-scale changes. Second, the residual trend method, though robust, implicitly attributes all non-climatic trends to “human activities,” a broad category encompassing heterogeneous socioeconomic, political, and managerial factors [45]. Our analysis lacks direct, spatially explicit data on specific policies (e.g., payments for ecosystem services under the Grain-for-Green Program), agricultural inputs (e.g., fertilization, irrigation intensity), or demographic shifts (e.g., rural–urban migration). This makes it difficult to distinguish the relative contributions of specific types of human activity — for instance, ecological restoration versus urban encroachment — using the residual approach alone. While we have mitigated this limitation by cross-validating the residual-based anthropogenic signal against independently observed NDVI_GS_ trend patterns and the known spatial distribution of major human activity types, a direct quantitative disaggregation of human activity subtypes remains beyond the scope of the current study. Incorporating such datasets through spatially econometric models would strengthen causal inference and enable a more nuanced attribution of anthropogenic effects. Third, the static classification of soil types does not account for potential changes in soil properties over the 24-year period, which could influence long-term vegetation productivity [46]. Finally, future studies should explore the coupling effects of extreme climate events (e.g., droughts, heatwaves) with human interventions on vegetation resilience, a critical aspect for climate adaptation planning [47].

## 5. Conclusions

This study integrates long-term MODIS NDVI time series with trend detection, partial correlation, GeoDetector analysis, and residual-based attribution to characterize vegetation dynamics and their drivers in Henan Province from 2000 to 2023. Vegetation greenness exhibits an overall improvement but with pronounced spatial heterogeneity across major river basins, indicating that regional-scale greening coexists with localized degradation hotspots under different physiographic and land-use settings. The results further indicate that the hierarchy of controls on NDVI_GS_ trends is basin dependent: topography–soil gradients and their interaction effects provide a strong environmental template in mountainous basins, whereas climate sensitivity and anthropogenic impacts become more prominent over intensively managed plains.

These findings advance current understanding of vegetation dynamics in coupled natural–human systems by demonstrating that spatial stratified heterogeneity and factor interactions are essential for explaining basin-resolved NDVI_GS_ trend variability, rather than relying on single-factor or spatially homogeneous assumptions. The combined diagnostic framework also complements existing greening China narratives by clarifying how basin-specific hydrothermal contexts modulate climate–vegetation coupling, and by isolating the net anthropogenic signal that tends to promote vegetation improvement while remaining spatially uneven. From a practical perspective, the mapped promoting and inhibiting anthropogenic effects provide actionable guidance for differentiated basin management: consolidating restoration gains where ecological interventions are effective, while prioritizing targeted mitigation in fragmented inhibition hotspots linked to urban expansion and intensive land use.

Future work should incorporate higher-resolution time series (e.g., Sentinel-2/Landsat), phenology- and productivity-related indicators, and spatially explicit socioeconomic and land-use intensity datasets to strengthen causal attribution and better resolve human–climate interactions, including the role of extreme events in shaping vegetation resilience. Overall, this study provides a basin-explicit, multi-method framework for diagnosing vegetation change and its drivers, offering a robust scientific basis for climate-adaptive ecosystem management and spatially targeted ecological planning in Henan Province.

## Supporting information

S1 File Raw remote sensing and meteorological raster datasets (RAR archive). Contains all original TIFF files used in this study, including vegetation indices (2000–2023) and meteorological driving factors for Henan Province.
(RAR)

S2 File Source code for driving factors analysis (RAR archive). Contains code for correlation analysis, Theil-Sen trend analysis, and geographically weighted regression.
(RAR)

S3 File Theil-Sen trend and residual result rasters (RAR archive). Contains TIFF files of Theil-Sen slope estimates, significance test results, and regression residuals.
(RAR)

S4 File Summary statistics of driving factors analysis (RAR archive). Contains detailed statistical metrics.
(RAR)
